# Supplementary material for: Tumour-elicited neutrophils engage mitochondrial metabolism to circumvent nutrient limitations and maintain immune suppression
Source: Nat Commun. 2018 Nov 30;9:5099. doi: 10.1038/s41467-018-07505-2 (PMC6269473; doi:10.1038/s41467-018-07505-2)
Supplement: Supplementary file 1 — Supplementary Information [file 41467_2018_7505_MOESM1_ESM.pdf]

Tumour-elicited neutrophils engage mitochondrial metabolism to circumvent nutrient limitations and maintain immune suppression

Rice et al.

Supplemental figure 1

Identification of bone marrow derived Immature c-Kit$^+$ neutrophils

a) Representative flow cytometry gating strategy of C57 bl-6 mouse bone marrow for Ly-6G$^+$ neutrophils prior to CXCR2 and c-Kit gating. **b)** Percentage of immature neutrophils in bone marrow subsets as determined by scoring of unsegmented nuclei with an immature chromatin pattern. Data (n=2) represents two similar experiments. **c)** Representative flow cytometry of Ly-6C and CD115 expression in c-Kit$^+$ (red) and c-Kit$^-$ (blue) neutrophils compared to monocytes. **d)** Representative flow cytometry of CD62L, CD11b and CXCR4 expression in c-Kit$^{+/-}$ neutrophils compared to Ly6G negative cells. **e)** Representative flow cytometry gating strategy of C57 bl-6 mouse bone marrow for Ly-6G$^+$, c-Kit$^-$/CXCR2$^+$ and c-Kit$^+$/CXCR2$^-$ neutrophils. All error bars display mean±SEM.

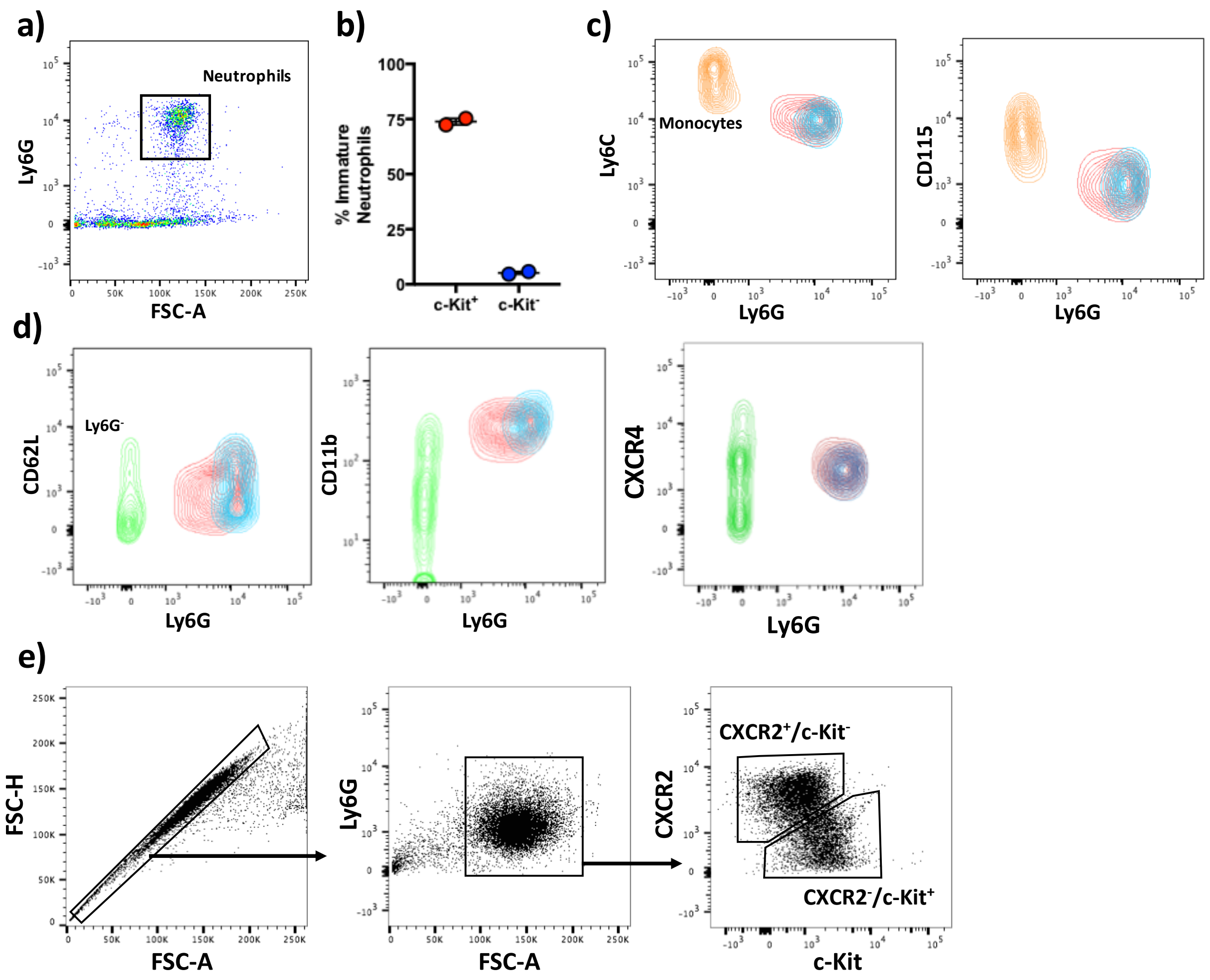

Supplemental figure 2

Immature bone marrow resident neutrophils have an altered metabolic phenotype

a) Representative flow cytometry gating strategy of C57 bl-6 mouse bone marrow for Ly-6G$^+$, c-Kit$^+$ and c-Kit$^-$ neutrophils and Ly-6G^{int} c-Kit$^+$ cells. **b)** Representative mitochondrial stress test of the cell populations identified in representative flow cytometry. **c)** Quantification of mitochondrial spare respiratory capacity from mitochondrial stress tests in supplemental Fig. f. Data shown represents one experiments (Ly-6G$^+$ n=5, Ly-6G^{int} n=3). Data were analysed by one way-ANOVA with Dunnett's multiple post-tests indicated. **d)** Representative nuclear morphology of Ly-6G^{int} c-Kit$^+$ cells following cytospin and Romanowsky staining. Scale bar represents 10μm. **e)** Representative flow cytometry histograms for Ly-6G, Ly-6C and CD115 staining in bone marrow resident populations. **f)** Quantification of flow cytometry staining for Ly-6G, Ly-6C and CD115 staining in bone marrow resident populations. Data shown represents one experiments (n=3) and were analysed by one way-ANOVA with Dunnett's multiple post-tests indicated. **g)** Western blotting of cytosolic (CYT) and mitochondrial (MIT) isolates for: aconitase 2 (ACO2), anti-lipoate to detect pyruvate dehydrogenase (PDH2-E2) and α-ketoglutarate dehydrogenase (αKGDH-E2), NDUFS1 (complex I) and succinate dehydrogenase B (SDHB) (complex II). Controls are TUB = β-tubulin, TOM20, a mitochondrial membrane protein, and cytosolic (c-HSC20) or mitochondrial HSC20. Data shown represents two independent experiments (n=3). **h)** Mitochondrial DNA content was measured as a ratio 16S/Actinβ as measured by RT-PCR after treatment of c-Kit$^-$ and c-Kit$^+$ with SCF (20ng/ml) for 12 hours. Data (n=4) from two independent experiments was analysed by Students t-test. **i)** In-gel assays for mitochondrial complex I, II and IV activities in c-Kit$^{+/-}$ neutrophils. * indicates an unidentified NADPH utilizing enzyme. **j)** Heat-map of metabolite peak areas measured by gas chromatography-mass spectrometry as defined by log2 of difference from the mean. Data shown were significant by students t-test (n=5 per group from one experiment). *p* values *<0.05 ** <0.01 ***<0.001. All error bars display mean$\pm$SEM.

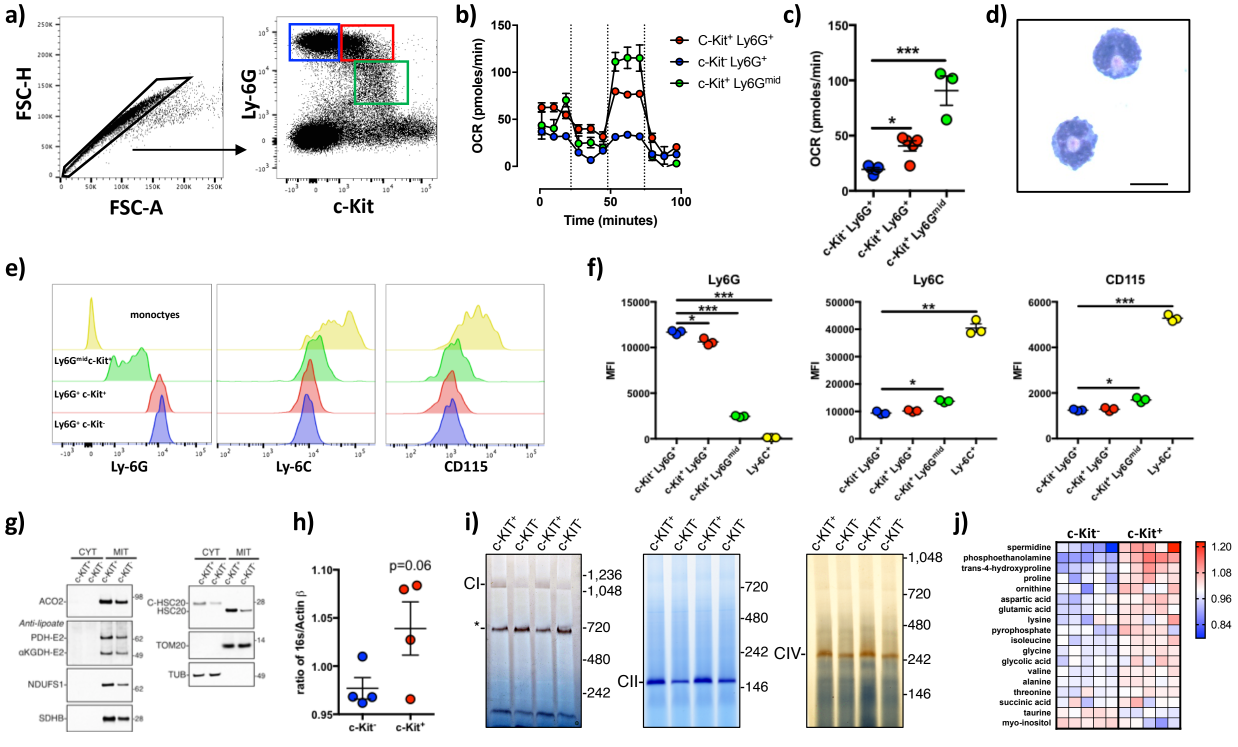

a)
FSC-H
FSC-A
Ly-6G
c-Kit
b)
OCR (pmoles/min)
Time (minutes)
C-Kit⁺ Ly6G⁺
c-Kit⁻ Ly6G⁺
c-Kit⁺ Ly6Gᵐⁱᵈ
c)
OCR (pmoles/min)
***
*
c-Kit⁻ Ly6G⁺
c-Kit⁺ Ly6G⁺
c-Kit⁺ Ly6Gᵐⁱᵈ
d)
e)
monoctyes
Ly6Gᵐⁱᵈc-Kit⁺
Ly6G⁺ c-Kit⁺
Ly6G⁺ c-Kit⁻
Ly-6G
Ly-6C
CD115
f)
Ly6G
Ly6C
CD115
MFI
c-Kit⁻ Ly6G⁺
c-Kit⁺ Ly6G⁺
c-Kit⁺ Ly6Gᵐⁱᵈ
Ly-6C⁺
g)
CYT
MIT
ACO2
Anti-lipoate
PDH-E2
αKGDH-E2
NDUFS1
SDHB
C-HSC20
HSC20
TOM20
TUB
h)
p=0.06
ratio of 16s/Actin β
c-Kit⁻
c-Kit⁺
i)
c-KIT⁺
c-KIT⁻
CI-
*-
CII-
CIV-
1,236
1,048
720
480
242
146
j)
c-Kit⁻
c-Kit⁺
spermidine
phosphoethanolamine
trans-4-hydroxyproline
proline
ornithine
aspartic acid
glutamic acid
lysine
pyrophosphate
isoleucine
glycine
glycolic acid
valine
alanine
threonine
succinic acid
taurine
myo-inositol
1.20
1.08
0.96
0.84

Supplemental figure 3

Neutrophil mitochondria facilitate free radical production

a) Area under the curve (AUC) oxygen consumption rates (OCR) of early (0-55 minutes) and late (55-306 minutes) respiratory burst following pre-treatment with indicated inhibitor and phorbol 12-myristate 13-acetate (PMA) stimulation. Data (n=6) from two independent experiments (quantification from figure 2a). Data were analysed by two-way ANOVA with Dunnett's multiple post-tests indicated. **b)** AUC of PMA stimulated respiratory burst following a second treatment of indicated inhibitor (post) and pre-treatment with indicated inhibitor (pre). Data (n=5) from two independent experiments (Quantification from figure 2b) and were analysed by paired t-test. **c)** AUC of PMA stimulated respiratory burst post treatment with etomoxir and pre-treatment with 2DG. Data (n=5) is representative of two independent experiments (quantification from figure 2d). Data were analysed by unpaired t-test. **d)** AUC of PMA stimulated respiratory burst post treatment with indicated inhibitor and pre-treatment with 2DG. Data (n=5) and is from two independent experiments (quantification from figure 2f). Data were analysed by two way-ANOVA with Dunnett's multiple post-tests indicated. **e)** AUC of PMA stimulated respiratory burst post treatment with oligomycin with and without Carbonyl cyanide-*4*-(trifluoromethoxy)phenylhydrazone (FCCP) and pre-treatment with 2DG. Data (n=4) and represents a single experiment (quantification from figure 2g). Data were analysed by two way-ANOVA with Dunnett's multiple post-tests indicated. **f)** Representative flow cytometry histograms of 2',7'–dichlorofluorescin diacetate (DCFDA) and aminophenyl fluorescein (APF) intensity in bone marrow derived neutrophils from wild type and NOX null ($p47^{-/-}$) mice following 20min stimulation with PMA. **g)** Basal mitochondrial OCR from $p47^{-/-}$ bone marrow neutrophils following stimulation as indicated. Data is representative of 3 independent experiments. Data were analysed by two-way ANOVA with Tukey's multiple comparison post-tests (Control v PMA **, Control v 2DG PMA ***, PMA v 2DG PMA ***). Concentrations were: rotenone (100nM), atpenin (1µM), antimycin A (1µM), 2-DG (100mM), PMA (1µg/ml), etomoxir (100µM), FCCP (660nM) and oligomycin (1.26µM), DCFDA (5µM), APF (5µM). *p* values ** <0.01 ***<0.001. All error bars display mean±SEM.

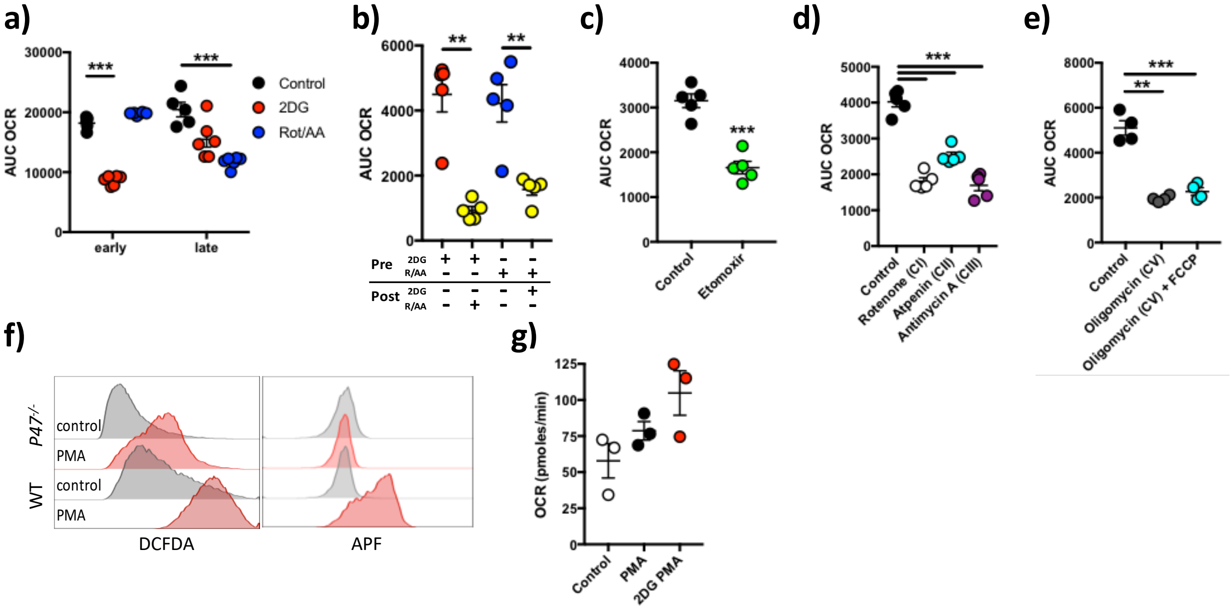
a)
30000
20000
10000
0
AUC OCR
***
***
Control
2DG
Rot/AA
early
late
b)
6000
4000
2000
0
AUC OCR
**
**
Pre
2DG
R/AA
Post
2DG
R/AA
c)
4000
3000
2000
1000
0
AUC OCR
***
Control
Etomoxir
d)
5000
4000
3000
2000
1000
0
AUC OCR
***
Control
Rotenone (CI)
Atpenin (CII)
Antimycin A (CIII)
e)
8000
6000
4000
2000
0
AUC OCR
***
**
Control
Oligomycin (CV)
Oligomycin (CV) + FCCP
f)
P47-/-
WT
control
PMA
control
PMA
DCFDA
APF
g)
125
100
75
50
25
0
OCR (pmoles/min)
Control
PMA
2DG PMA

Supplemental figure 4

c-Kit+ neutrophils utalise their mitochondrial in response to zymosan

a) Relative area under the curve (AUC) of C57BL/6 bone marrow c-Kit$^+$ and c-Kit$^-$ subsets neutrophils following stimulation with zymosan in the presence of rotenone (100nM) or antimycin A (1µM) compared to vehicle control. Data (c-Kit$^+$ n=3, c-Kit$^-$ n=4) from 4 independent experiments and were analysed by two-way ANOVA with Sidak's multiple comparisons represented on graph. *p* values ** <0.01 ***<0.001. All error bars display mean±SEM.

a)

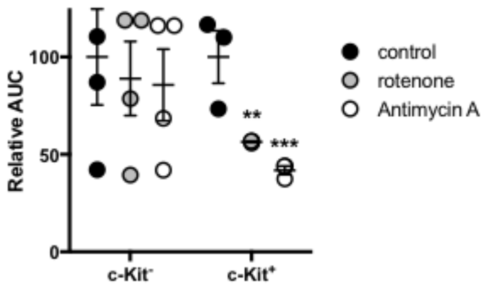

Supplemental figure 5

4T1 tumours elicit c-Kit^{+} neutrophils with mitochondrial metabolism

a) Neutrophil counts from naïve and 4T1 tumour-bearing Balb-c mouse tissues. Data are from two independent experiments and were analysed by two-way ANOVA with Tukey's post-test indicated on graph (n=5). **b)** Image of a 4T1 (14 day) tumour section with hematoxylin and eosin stain, examples of neutrophils are highlighted by arrows. Image is representative of multiple photographs from independent experiments. **c)** Flow cytometry plots displaying increased c-Kit expression in Ly-6G^{+} splenic and circulating neutrophil populations from naïve and 4T1 bearing mice. Boxes indicate c-Kit^{+} neutrophils. Data shown represents multiple experiments. **d)** Representative flowcytometry histograms for Ly6G, CD62L, CXCR2 and c-Kit expression in splenic neutrophils from naïve and 4T1 bearing mice. **e)** Percentage of immature nuclear morphology in c-Kit$^{+/-}$ neutrophils from 4T1 bearing mice spleens. **f)** Representative mitochondrial stress test of neutrophils derived from blood of 4T1 bearing mice compare to healthy naïve controls (left). Quantification of basal and maximal OCR (n=3) from three independent experiments (right). Data were analysed by two way-ANOVA with Sidak's multiple post-tests indicated. **g)** Representative Extracellular acidification rates (ECAR) and oxygen consumption rates (OCR) traces from a mitochondrial stress test of neutrophils derived from bone marrow and the tumour site in 4T1 bearing mice. **h)** In-gel assays for mitochondrial complex II and IV activities in splenic neutrophils from naïve and 4T1 bearing mice. **i)** Western blotting of mitochondrial complex III subunit (UQCRFS1) with voltage-dependent anion channel (VDAC) as a loading control in splenic neutrophils from naïve and 4T1 bearing mice. Data (e and f) (n=2) from a single experiment. **j)** Heat-map of metabolite peak areas measured via gas chromatography-mass spectrometry as defined by log2 of difference from the mean (n=5 per group from one experiment). *p* values *<0.05 ***<0.001. All error bars display mean±SEM.

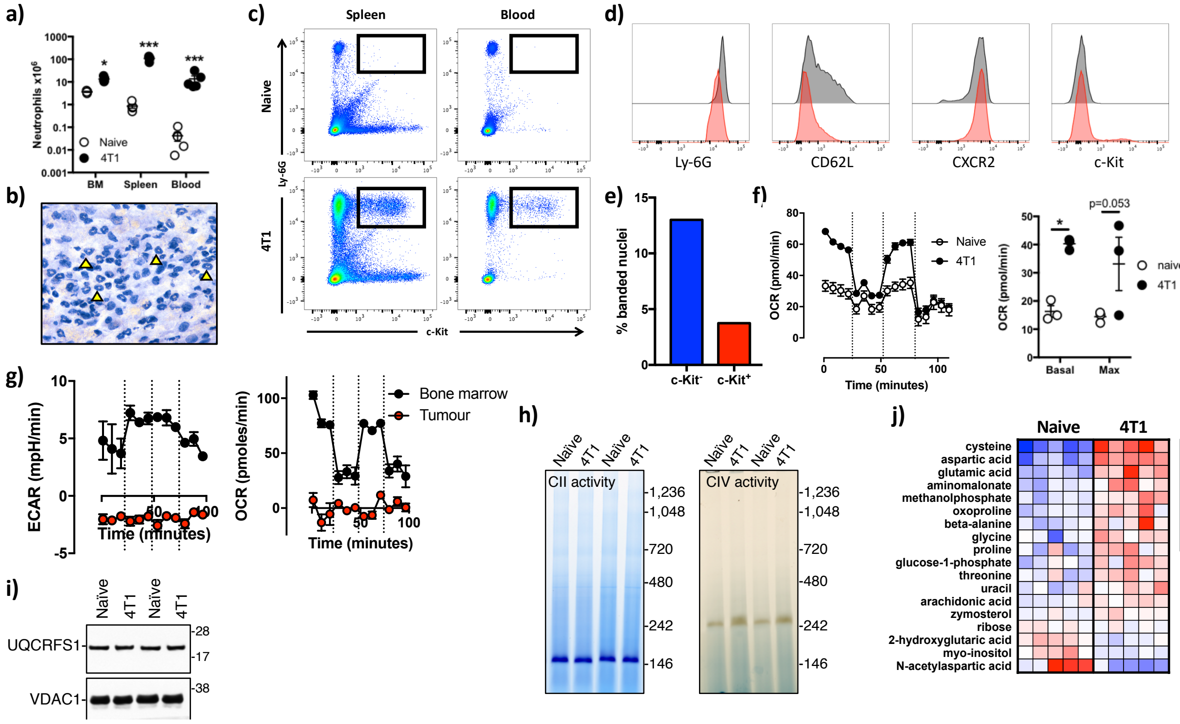

Supplemental figure 6

4T1 tumours produce SCF but do not express c-Kit

a) SCF production from 4T1 cultures *in vitro* compared to media alone. Data is representative of two independent experiments. Combined data from independent experiments were analysed by two-way ANOVA (4T1 n=5 media n=4) p<0.001. **b)** Representative flow cytometry histograms of anti-c-Kit (red) compared to isotype controls (black) in 4T1 cells derived from in vitro cultures. **c)** Non-homogenous end joining (NHEJ) induced mutation rates from CRIPR candidates 50-1 and 51-1 and a non-transformed 4T1 control. **d)** *in vitro* SCF production from 4T1 cell lines which received CRISPR plasmid. Data is from two independent experiments and were analysed by two way ANOVA (4T1 n=5, 50-1 and 51-1 n=4) p<0.001. All error bars display mean±SEM.

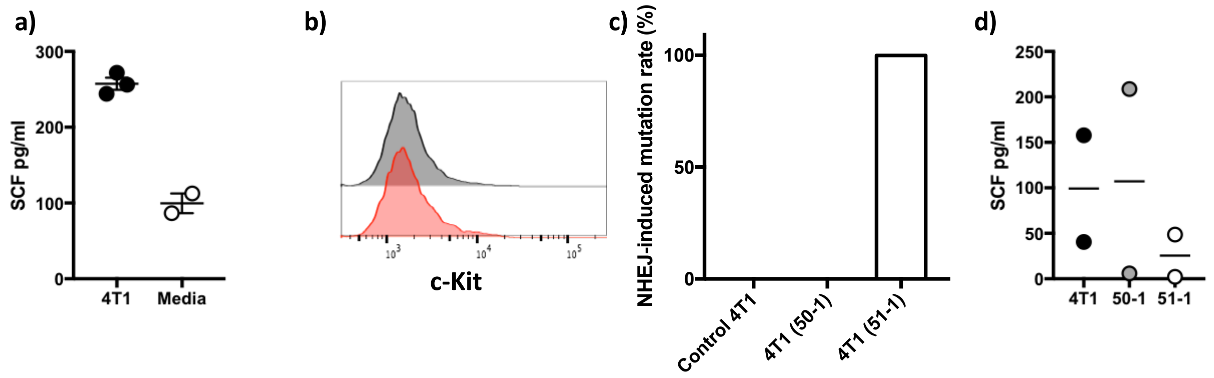

Supplemental figure 7

Activated neutrophils inhibit T cell function

a) $CD3^+$ T cell viability as measured by non-permeable Sytox DNA staining following 3 day culture alone (T cells), co-culture with unstimulated neutrophils (control) or neutrophils pre-stimulated with PMA. Data (n=3) are from a single experiment and were analysed by one-way ANOVA with Tukey's multiple comparisons indicated on graph. **b)** Representative flow cytometry histograms of Cell Trace violet dye used to measure rounds of proliferation under culture conditions described in (a) (left). Proliferation index of $CD3^+$ T cells as calculated from cell trace violet histograms (right). Data (n=3) are from a single experiment and were analysed by one-way ANOVA with Tukey's multiple comparisons indicated on graph. **c)** Interferon-γ (IFN-γ) production from T cell co-cultured with unactivated neutrophils (control) or neutrophils pre-stimulated with PMA (PMA). Data (n=3) are from a single experiment and were analysed by paired t-test. **d)** $CD8^+$ T cell killing following co-culture with splenic neutrophils. Neutrophils were pre-activated with PMA in the presence of 2-deoxy glucose (2DG, 100mM), data is relative to PMA pre-treatment of neutrophils alone. Data (naïve n=4, 4T1 n=3) is from two independent experiments. *p* values ** <0.01 ***<0.001. All error bars display mean±SEM.

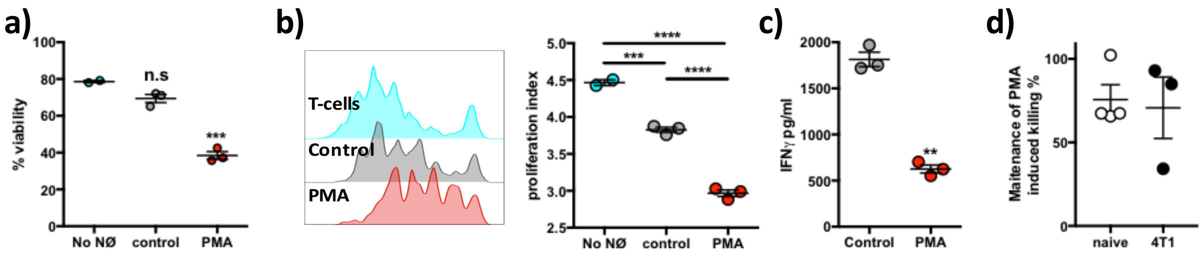

Supplementary Table 1
Antibodies used for flow cytometry

Antibody target	Fluorophore	Company and Catalogue number	Dilution
Mouse Ly6G	Pacific Blue	Biolegend 127612	1/100
Mouse Ly6G	APC	Biolegend 127614	1/100
Mouse Ly6C	PerCP	Biolegend 128028	1/100
Mouse CXCR2	FITC	Biolegend 149310	1/100
Mouse c-Kit	APC	Biolegend 135108	1/50
Mouse c-Kit	PE	Biolegend 135106	1/50
Mouse CD115	APC	Biolegend 135510	1/100
Mouse CD62L	Fitc	Biolegend 104406	1/100
Mouse CD4	APC	BD Bioscience 553051	1/100
Mouse CD8a	PER CP	BD Bioscience 553036	1/100
Human CD10	PerCP/Cy5.5	Biolegend 312216	1/30
Human CD14	FITC	BD Bioscience 347493	1/30
Human CD15	APC	Biolegend 301908	1/30
Human CD16	PerCP/Cy5.5	BD Bioscience 338426	1/30
Human CD16	PE	BD Bioscience 561313	1/30
